# Optimizing Care Pathways from Screening/Detection to Survivorship for Early Age Onset Cancer Patients in Canada

**DOI:** 10.3390/curroncol33070377

**Published:** 2026-06-23

**Authors:** Michael J. Raphael, Darren R. Brenner, Tanya Chawla, Trudy Matwiy, Stuart Peacock, Robby Spring, Perri R. Tutelman, Eva Villalba, Cassandra Macaulay, Filomena Servidio-Italiano

**Affiliations:** 1 Sunnybrook Health Sciences Centre, Toronto, ON M4N 3M5, Canada; michaelj.raphael@sunnybrook.ca; 2 Departments of Oncology and Community Health Sciences, Cumming School of Medicine, University of Calgary, Calgary, AB T2N 1N4, Canada; darren.brenner@ucalgary.ca; 3 Joint Department of Medical Imaging, University of Toronto, Mount Sinai Hospital, Toronto, ON M5G 1X5, Canada; tanya.chawla@sinaihealth.ca; 4 Crossfield Clinic, Crossfield, AB T0M 0S0, Canada; primarycare@albertanps.com; 5 BC Cancer Research Institute, Vancouver, BC V5Z 1L3, Canada; speacock@bccrc.ca; 6 Independent Patient Advocate and Healthcare Strategist, Toronto, ON, Canada; springrobby@gmail.com; 7 Department of Psychology, University of Calgary, Calgary, AB T2N 1N4, Canada; perri.tutelman@ucalgary.ca; 8 Quebec Cancer Coalition, Saint-Lambert, QC J4P 1X2, Canada; evav@coalitioncancer.com; 9 Colorectal Cancer Resource & Action Network (CCRAN), Toronto, ON M4W 3E2, Canada; cassandra.m@ccran.org

**Keywords:** early age onset cancer, survivorship, screening, fertility

## Abstract

The fifth annual pan-tumour Early Age Onset Cancer (EAOC) Symposium, held in November 2025 and organized by the Colorectal Cancer Resource & Action Network (CCRAN), convened clinicians, researchers, policymakers, patients, and caregivers to address the rising incidence of cancers in individuals under 50 years. In addition to discussions around diagnostic and therapeutic advances for patients with late-stage disease, content centered on addressing critical gaps along the EAOC care continuum, including (i) diagnostic delays related to limited awareness and suboptimal primary care pathways, (ii) screening eligibility criteria for colorectal cancer (CRC) that no longer reflect current disease epidemiology, and (iii) insufficient age-appropriate infrastructure to meet the EAOC population’s unique unmet needs with respect to psychosocial support, fertility counseling, financial navigation, and survivorship planning. The symposium generated consensus recommendations such as the embedding of EAOC education into medical training curricula to increase the index of suspicion of EAOC in primary care, lowering the CRC screening age to 45 years to match this population’s rising disease incidence, and expanding multidisciplinary adolescent and young adult (AYA) and EAOC programs—including through the use of virtual models—to ensure that patients receive coordinated, comprehensive, equitable and age-appropriate care across the country.

## Simple Summary

The Colorectal Cancer Resource & Action Network (CCRAN) held its fifth annual pan-tumour Early Age Onset Cancer (EAOC) Symposium in November 2025, focusing on the growing incidence of cancer among people under 50, and addressing major gaps in EAOC care. Key concerns included delayed diagnoses attributed to limited EAOC awareness and suboptimal primary care pathways, outdated colorectal cancer (CRC) screening criteria that do not reflect the rising rates of disease in the EAOC population, and inadequate age-appropriate support services. Participants highlighted the unique, unmet needs of the EAOC population related to psychosocial support, fertility counseling, financial navigation, and survivorship planning. The symposium resulted in several recommendations including integrating EAOC education into medical training, lowering the CRC screening age to 45 years, and expanding multidisciplinary adolescent and young adult (AYA) and EAOC programs, including virtual care models, to improve equitable, coordinated, and comprehensive support nationwide.

## 1. Introduction

In recent years, the incidence of early age onset cancer (EAOC) diagnosed in individuals under 50 years of age has risen markedly in Canada [1]. This disturbing epidemiological trend is most prominently driven by gastrointestinal cancers and, in particular, colorectal cancer (CRC), but extends to 14 other tumour types including breast and endometrial cancers [1,2,3,4,5].

EAOC tends to be diagnosed at an advanced stage due to the lack of knowledge of young people and healthcare providers (HCPs) regarding presentations of cancer in this population coupled with screening paradigms weighted towards older age groups, and tumour biology that is often more aggressive than cancers in older patients. Combined with persistent gaps in care—including limited access to diagnostics and a lack of specialized programs for younger patients that address the significant psychological, financial, fertility, and care-access barriers, they face—a perfect storm is emerging across the EAOC care trajectory. Recognizing these challenges, in 2021, the Colorectal Cancer Resource & Action Network (CCRAN) initiated an annual symposia series dedicated to confronting the rising rates of cancer in the under-50 population and to supporting this cohort’s needs [6]. Bringing together clinicians, researchers, policy professionals, patients, and caregivers, the 2025 pan-tumour EAOC Symposium provided a structured forum to explore the system-level challenges that impede timely, equitable, high-quality, and age-appropriate care for individuals with EAOC (Appendix A, Appendix B, Appendix C and Appendix D).

Symposium content included a review of recent therapeutic advances pertinent to EAOC patients presenting at advanced stages of disease. The field of immunotherapy continues to evolve; in patients with recurrent or metastatic endometrial cancer, data from the RUBY trial demonstrated that the combination of an immune checkpoint inhibitor with standard-of-care chemotherapy increased overall survival, with the highest reduction in mortality observed in mismatch repair-deficient/microsatellite instability-high (dMMR/MSI-H) sub-populations [7]. For those with proficient MMR (pMMR) endometrial cancer, the results from the DUO-E II trial found that the addition of a poly(ADP-ribose) polymerase (PARP) inhibitor conferred overall survival benefits, opening possible treatment avenues for this population [8]. In microsatellite stable (MSS) CRC, phase 1 and 1b studies found that the dual approach of botensilimab and balstilimab (BOT/BAL), two immune checkpoint inhibitors, yielded improvements in disease control and overall survival, representing a potentially critical shift in the treatment landscape for a population with historically limited options [9,10].

Genomic medicine featured prominently in symposium discussions, with key findings presented from the first national costs and benefits analysis of universal comprehensive genomic profiling (CGP) for five tumour types in the metastatic setting. With CGP, a diagnostic tool that analyzes the genomic sequence of tumours to identify actionable mutations and guide treatment decisions, the model estimated an increase of 3440 life-years with $87M–134M of potential healthcare system savings over a six-year time horizon [11,12]; these results infer significant potential for CGP to improve the cancer journey for EAOC patients.

A primary focus of the 2025 EAOC Symposium was to close gaps in EAOC patient care from screening/detection to survivorship. Sessions were dedicated to promoting earlier detection of EAOC through strategies to support primary care providers (PCPs) in diagnosis, the value of lowering the screening age for colorectal cancer (CRC), and enhancing the treatment and survivorship journey for EAOC patients by modifying traditional models of care to accommodate the needs of this younger patient population and their care partners. This narrative synthesis and consensus report summarizes the key scientific presentations, multi-disciplinary expert discussions, and emerging priorities across the EAOC care continuum. In building this report, the coauthors synthesized the discussion and recommendations generated during the symposium and created four calls to action with the aim of informing ongoing efforts to improve outcomes for this growing patient population, through optimal clinical practice, health systems planning, enhanced education and awareness, and policy development in Canada.

## 2. Building a Primary Care System That Elevates EAOC Awareness and Screening/Detection

Having timely and efficient primary care pathways is essential for identifying EAOC at early stages, thereby reducing diagnostic delays and advancing upstream intervention through specialty care in the initial phases of the EAOC patient journey. Symposium panelists discussed strategies to enhance the primary care system’s readiness for early detection of EAOC.

### 2.1. Supporting PCPs in Helping to Increase Index of Suspicion for EAOC

As the first point of care and entry into the healthcare system for most Canadians, PCPs occupy a pivotal role in EAOC screening/detection and diagnosis. However, patient panelists described diagnostic experiences whereby, due to their age and otherwise healthy status, their symptoms were repeatedly dismissed by their PCP. This pattern is corroborated by prior literature [13], and is not reflective of individual clinicians, but rather a healthcare system built on the epidemiological realities of decades ago, leaving it ill-equipped to manage the current EAOC crisis. Compounding this, no standardized specialist referral pathway exists for PCPs managing a patient with suspected EAOC. The process of initiating a referral, navigating a decline, awaiting a call-back and re-starting the process with a second specialist is burdensome and time-consuming, leading to delayed care as well as disease progression [14,15].

Data were presented from a 2021 Quebec study examining pre-diagnostic pathways in lung cancer; those for whom PCP visits were included prominently in their diagnostic journey, took twice as long to receive a referral (45 vs. 22 days), and were diagnosed with more advanced disease (65% vs. 50%), compared to those primarily receiving care through walk-in clinics and ERs [16]. While not specific to EAOC, this highlights the urgent need for greater EAOC awareness within frontline care settings, as well as optimal standardized referral pathways.

To strengthen the primary care system’s readiness for early detection of EAOC, enhancing EAOC awareness and risk recognition should be initiated at early stages of clinical training, through expanded curricula in both medical schools and nursing schools. Patient organizations can further support education efforts by developing and disseminating practical tools and evidence-based materials for PCPs, to educate on EAOC epidemiology, risk factor identification and symptom patterns. This coordinated effort will be essential to closing the current gap between symptom onset and timely diagnosis in the EAOC population.

An additional challenge with timely detection and diagnosis of EAOC is that nearly one-fifth of the Canadian population does not have a PCP [17]. In Alberta, the Nurse Practitioner (NP) Primary Care Program is helping to address this gap, providing funding for NPs to practice comprehensive primary care and operate their own independent clinics, or practice independently in team-based care settings. Under the recently revised Canada Health Act, each province and territory will need to provide coverage for medically necessary services provided by NPs; this shift is intended to help offset family physician shortages, and attach more Canadians to PCPs [18].

### 2.2. Balancing the Benefits of Early EAOC Detection with System-Level Realities

Symposium panelists discussed the individual-level and system-level benefits of early detection of EAOC, citing evidence from both United Kingdom and Canadian studies on how structured screening programs can meaningfully shift the distribution of cancer diagnoses toward less advanced disease. Data from the UK Lung Cancer RCT Pilot Screening Trial showed that low-dose CT screening among high-risk individuals identified lung cancer in 2.1% of participants, of whom 66.7% presented with Stage I and 19% with Stage II disease, enabling surgical resection in 83.3% of cases and yielding 137 additional life-years across 42 patients, and improved quality of life [19]. Similarly, a Cancer Care Ontario (CCO) analysis of 48,000 women diagnosed with breast cancer between 2010 and 2017 found that women whose cancer was detected by screening were diagnosed at an earlier stage and had lower mortality compared to those identified outside of screening [20]. Critically, earlier detection was associated with substantial healthcare system cost savings attributable to less aggressive treatment needed and reduced utilization of resources such as drug therapies, oncology beds, ICU and palliative services [19].

Panelists acknowledged that despite this evidence, policymakers must consider competing priorities when deciding on new screening initiatives in the context of finite funding. A value-based healthcare (VBHC) framework—one that prioritizes improvement of total health outcomes relative to costs (and resources)—was proposed as a constructive lens through which to evaluate the systemic return on investment of earlier detection. By positioning screening as the starting point for optimization of the care pathway and recognizing prevention and early detection as more cost-effective strategies for achieving better outcomes, this approach highlights the value of investing in screening and early detection. Existing Canadian programs aligned with this model include HPV testing for detection of pre-cancerous cervical lesions and breast cancer screening for individuals aged 40 and older. Panelists emphasized that further evidence is needed to make a compelling case to decision-makers for accelerating early detection and diagnosis; to this end, in collaboration with the Quebec Cancer Coalition and supported by CCRAN, Montreal’s Jewish General Hospital successfully incorporated VBHC into a quality improvement initiative for CRC patients, improving outcomes and reducing use of healthcare resources and related costs [21]. This VBHC project is scaling to other tumour types and is currently examining the impact of reducing time from clinical suspicion through diagnosis to treatment initiation on patient outcomes in lung cancer, to quantify the value of earlier intervention at the University Institute of Cardiology and Pulmonology of Quebec.

## 3. Critical Need for Earlier Screening/Detection of Early Age Onset Colorectal Cancer (EAOCRC)

There has been an alarming rise in the incidence of EAOCRC among young Canadians, and nearly every province now equals or exceeds the US rates of EAOCRC in 2015–2016, which ultimately prompted the country to sound the alarm and lower the CRC screening age of eligibility [22,23]. During the symposium, panelists explored strategies to overcome the systemic, jurisdictional, and access-related challenges that plague the first stages of the care pathways for EAOCRC, hindering equitable and earlier screening/detection.

### 3.1. Lowering the Screening Age for CRC to Align with the New Epidemiology of This Disease

The lowering of the screening age for breast cancer to 40 years across provinces and territories in Canada was precipitated by emerging epidemiology, cost modeling data, and sustained patient advocacy, and panelists described the growing urgency to apply the same strategy to advance policy towards addressing current gaps in early detection of CRC in the population under the age of 50 years. A 2026 Canadian modeling study demonstrated that implementing fecal immunochemical test (FIT)-based index screening at age 45, rather than 50 years, with follow-up colonoscopy for positive tests, would be associated with a reduction of 15,070 CRC cases and 6100 deaths between 2025 and 2071 [24]. These were accompanied by an overall decrease in CRC-related healthcare system costs with the higher upfront investment in screening negated by the decrease in the overall costs of CRC management due to earlier-stage diagnosis. This analysis reinforced a fundamental principle regarding the value of screening: preventing CRC cases (or detecting them at an earlier, more treatable stage) generates both survival benefits as well as healthcare system efficiencies.

Panelists presented corroborating evidence from a 2024 Kaiser Permanente study showed that the CRC positivity rates in individuals aged 45–49 years are nearly identical to those in the 50–54 age group, further supporting that lowering the screening age is an appropriate resource utilization decision [25]. In the US, this screening change has successfully detected cases in asymptomatic individuals that would have otherwise not been identified until the cancer had progressed to a more advanced and less treatable stage.

Beyond its direct clinical impact, lowering the screening age has carried the important secondary benefit of heightening awareness of EAOCRC among PCPs, who are now more liable to consider CRC as a potential diagnosis when evaluating younger patients presenting with relevant symptoms. Together, epidemiological and modeling data build a compelling evidentiary foundation for EAOCRC screening reform in Canada, and have resulted in both Prince Edward Island and Ontario making recent decisions to lower their screening age from 50 to 45 years [26,27].

### 3.2. Creating the System Infrastructure to Handle Increased Screening Capacity

Despite the obvious benefits from a reduced CRC screening age, Canada’s single-payer public health system carries significant resource constraints that must be carefully considered, as provinces and territories balance the critical need for screening with timely access for symptomatic patients. Panelists discussed endoscopy capacity as a fundamental nationwide issue; private endoscopy clinics partially offset demand in large cities but are absent in smaller and rural regions [28]. Lowering the CRC screening age may inadvertently generate downstream consequences, diverting limited endoscopy clinic slots from symptomatic patients awaiting diagnosis.

To mitigate these competing demands, panelists identified several strategies to enable population-level screening of asymptomatic individuals, while preserving prioritized access for symptom-based testing:

- Establishing priority groups within screening eligibility will help ensure that high-risk individuals are assessed preferentially.
- Use of biomarker testing to predict the presence of adenomas that may identify high-yield scores can further refine patient prioritization.
- Allowing patients to self-refer for a FIT screening ensures that healthy low-risk patients are not proceeding directly to more invasive testing, and diverting resources from higher-risk populations.
- Finally, the integration of multidisciplinary care models—such as nurse-led endoscopy programs to facilitate scoping—can further expand capacity and alleviate system burden.

## 4. Supportive Care Needs of EAOC Patients from Diagnosis to Survivorship

Panelists focused on the persistent gaps in EAOC supportive care throughout the care continuum, and proposed collaborative strategies to address them, including those that have since been summarized in a 2026 environmental scan [29]. Given that care programs for the EAOC population are often also targeting adolescents and young adults (AYA, individuals between 15 and 39 years of age), the combined term *AYA/EAOC* is used throughout this section.

### 4.1. The Results of CCRAN’s National Pan-Tumour Patient Survey

During the symposium, the results of CCRAN’s 2025 National Pan-Tumour Patient Survey were presented to share key findings and identify patient priorities related to AYA/EAOC care pathways throughout treatment and survivorship. The online survey was disseminated to patients via CCRAN social media channels as well as through 21 patient organizations across Canada.

#### 4.1.1. Demographics

- A total of 143 respondents between 18 and 49 years of age completed the online survey.
- Most respondents were from Ontario (38%), British Columbia (20%) and Alberta (14%), reflecting the population sizes as well as the locations of major cancer centers.
- Multiple tumour types were represented, most commonly breast, CRC and blood.

#### 4.1.2. AYA/EAOC Clinic Access and Utilization

- In total, 83% of respondents did not have access to an AYA/EAOC clinic and the majority of patients believed access would have significantly or somewhat improved their care (45% and 41%, respectively).
- Of those with access to a dedicated clinic, half used the service regularly and 35% occasionally; the programs to support mental health, nutrition and fertility were most commonly used, aligned with the primary unmet needs identified by EAOC patients in traditional models of care.
- For 65%, the AYA/EAOC clinic was located at their treatment center; 29% utilized a virtual clinic, a helpful modality for jurisdictions without specialized EAOC care.
- Only 29% of referrals originated from oncologists, underscoring the absence of a standard referral pathway.

#### 4.1.3. Outcomes and Impact

- Those who received care at an AYA/EAOC clinic were less likely to feel socially disconnected, experience challenges with coordinating care, and report long wait times; however, they expressed unmet needs including support related to fear of relapse, employment, education and financial concerns.
- Patients seen at an AYA/EAOC clinic were less likely to have to coordinate their own care, than those in traditional care models (18% vs. 51%, respectively).
- Visible gaps exist across both models of care with respect to survivorship, particularly for women; only a minority of patients felt very well-supported in care coordination (26%), social re-integration (10%) and long-term side effect management (9%).
- Additionally, 79% did not receive a survivorship care plan, indicating a system-wide weakness.

The survey findings indicate the value to AYA/EAOC patients of dedicated models of care which despite growing need, are largely absent across Canada except in large urban hubs. As a result, patients receive non-specialized, fragmented care that they must coordinate on their own. This survey has various limitations that must be considered when interpreting the results, including the survey’s small sample size, the lack of representation across all provinces and territories as well as all tumour types, and the potential for both response bias and recall bias from respondents. Additionally, given that the survey dissemination strategy relied heavily on patient organizations, it is possible that responses may be more representative of patients that engage with these groups than those that do not. The results strongly highlighted the substantial gaps in clinical, psychosocial, and supportive care services experienced by AYA/EAOC patients across the cancer care pathway. Patients with access to tailored AYA/EAOC clinics reported improved care coordination and fewer challenges while receiving care, though unmet needs persist, particularly during survivorship. These gaps may be a result of resource and capacity restraints, and are indicative of opportunities to strengthen and standardize models to deliver age-appropriate care to patients along each stage from diagnosis to survivorship.

### 4.2. Opportunities for System-Level Improvements in AYA/EAOC Care

The findings from CCRAN’s 2025 National Pan-Tumour Patient Survey were validated by patient organization panelists as well-aligned with their AYA/EAOC members’ needs around sexual health and reduction in isolation. For many cancers, including CRC, pancreatic and prostate, younger patients do not see themselves reflected in the typical patient profile, contributing to loneliness [30,31,32]. Panelists discussed how this issue is magnified in underserved populations facing language barriers, limited health literacy and who may experience cultural stigmas around illness that prevent them from seeking care, and heighten isolation, further emphasizing the importance of cultural and inclusionary care models that incorporate peer/community support [33,34,35].

Models of care must be built with AYA/EAOC patients in mind, involving routine assessment of psychosocial and supportive care needs, and survivorship planning early in the treatment trajectory. Drawing on experiences from North America, Australia and the United Kingdom, the literature supports the development of AYA/EAOC clinics with embedded patient navigation, that prioritize the delivery of services including clinical trial enrollment and fertility counseling, within environments attuned to AYA/EAOC patients’ social and psychological needs [36].

The sustainability of these clinics is an overarching goal that may be facilitated by their transition from hospital-based to the community, or through a virtual model, under the auspices of local cancer centers that can share existing resources so that the responsibility of their operations is not borne by a single organization. Access and resource use may also be optimized by a pan-tumour approach to AYA/EAOC programming. However, for tumour types with larger AYA/EAOC populations, there may be benefits to having tumour-specific clinics to provide tailored support that addresses unique needs. For example, for patients with EAOCRC, treatments can result in lifelong issues with bowel dysfunction and ostomy care, carrying significant body image concerns that need support and handling from psychosocial and clinical perspectives [37].

Integrated research and evaluation are required to demonstrate the impact of the dedicated models of care on quality of life as well as system efficiencies, to inform programming and facilitate ongoing funding mechanisms.

#### 4.2.1. Sexual Health and Fertility

Cancer treatments can have a detrimental impact on sexual health, often causing pain, bleeding, erectile dysfunction, and other symptoms during intercourse, with significant decreases in libido [38]; such effects are not commonly discussed, despite guidance from groups including Cancer Care Ontario and the American Society of Clinical Oncology (ASCO) on addressing these common patient challenges [39,40,41]. The impacts of treatment can also extend to reproductive function, and while guidelines recommend that HCPs initiate early discussions with patients regarding their fertility options, this is not often prioritized due to other treatment-related concerns [38,41]. At a time when many AYA/EAOC patients have not previously contemplated family planning, they must quickly make decisions amid systemic barriers to accessing fertility support that often carry significant costs for which they have not prepared. Public funding for these services varies widely across the country, causing added stress and frustration for patients; a uniform approach is needed to cover common services for cancer patients such as egg or sperm freezing. Additionally, the silos of sexual health and oncology must open communication to provide comprehensive care regarding sexual function and fertility to younger cancer patients. While the onus should not be on the oncologist to explain reproductive options to patients, they should broach the topic (and perhaps indicate in the clinic notes that the conversation has been had) and connect them with AYA/EAOC clinics with collaborating fertility care teams, who can provide proactive counseling, including risk awareness and preservation strategies.

Online resources can further help patients understand possible changes in body image and changes in hormones post-treatment and their impact on sexual identity, function, and intimacy.

#### 4.2.2. Financial Support

Symposium speakers described the financial consequences of cancer as a significant, yet inadequately addressed, dimension of care for AYA/EAOC patients. A recent survey found that nearly one-quarter of Canadians diagnosed with cancer reported that they faced “substantial out-of-pocket costs”, yet many are diagnosed while establishing careers or pursuing education, and lack financial reserves to absorb the costly burden of illness [42]. Furthermore, individuals with AYA/EAOC are often diagnosed at an advanced stage, and are unable to work due to the impacts of their disease and treatments, with cascading effects on employment retention, housing stability and food security [42]. The long-term impact of this unexpected financial burden is devastating, with 40% of cancer patients reporting that their out-of-pocket costs during treatment cut into their retirement savings. Caregivers face additional systemic barriers, including restrictive workplace leave policies that hinder their ability to provide care. Programs exist across Canada to relieve the administration burden of applying for government initiatives and charities, but availability is inconsistent. Embedding financial counseling and resource navigation within dedicated AYA/EAOC clinics is essential to mitigating these hardships for patients, and supporting the long-term financial and psychosocial wellbeing of this population.

#### 4.2.3. Survivorship

Given the rise in EAOC and the significant oncological advancements that have extended survival, survivorship care is now of critical importance. Patient panelists discussed the secondary cancers and long-lasting physical impacts of EAOC and treatment including fatigue, cognitive impairment, body image changes, early menopause, bladder and bowel movement symptoms, and functional decline [43]. These challenges are intensified by psychological and social effects due to the patient feeling distanced from their peers due to their cancer journey, heightening feelings of isolation and altered identity. To address patients’ multidimensional survivorship needs, healthcare systems must evolve accordingly, to provide services—potentially via alternative models of care—designed to foster long-term wellbeing and psychological resilience. While community agencies aim to address and lessen some of these physical, psychological and social impacts, many programs are not tailored to younger patients. Initiatives such as BC Inspire are examining community driven approaches to improve the uptake of services among underserved populations, including South Asian communities. Strengthening partnerships across hospital, outpatient, and community settings is essential to ensuring that providers are informed about available support—including virtual programs and community-based resources—and can facilitate timely, appropriate referrals to meet the evolving needs of younger cancer survivors.

## 5. Conclusions

The 2025 EAOC Symposium yielded rich discussion on topics critical to the advancement of early detection of EAOC in Canada and the support of patients throughout the cancer continuum and into survivorship. A sixth symposium is being planned for November 2026, with the theme of *EAOC: Challenging Current Models of Care in Canada*, to make further progress in addressing the unique needs of the pan-tumour EAOC community.

From the symposium, multiple calls to action were generated (Table 1).

## Figures and Tables

**Table 1:** Calls to Action generated through synthesis of the 2025 EAOC Symposium discussions.

Domain	Calls to Action	Responsible Stakeholders
Education and Clinical Practice	Increased EAOC education for PCPs must be embedded into the curriculum of medical school and nursing school, and through ongoing continuing medical education (CME) training to facilitate their ability to detect EAOC in patients. Patient organizations should continue to promote awareness and education to PCPs about the rising rates of EAOC.	Patient organizations Clinician bodies Nursing bodies Medical/nursing school leadership
Policy	2. The eligibility criteria of average-risk CRC screening programs must be lowered to 45 years in provinces and territories across Canada to align with the changing epidemiology of the disease. Through changes including patient self-referral for FIT and the prioritization of colonoscopies for high-risk and symptomatic patients, the healthcare system can better adapt to accommodate the varying needs of the under-50 year population.	Provincial and territorial governments
Health Systems Planning and Clinical Practice	3. Funding must be allocated to specialized AYA/EAOC models of care across the country that involve multidisciplinary care teams to provide coordinated support with the psychosocial and financial burdens of cancer, fertility planning and survivorship care. Virtual models should be viewed as a viable option that overcomes many financial and logistical barriers and establishes consistency where in-person models are not feasible. Additionally, standard and effective referral pathways are needed to ensure that patients can avail themselves of this age-appropriate care model.	Federal government Provincial and territorial governments Hospitals
Education and Awareness	4. Increase awareness of AYA/EAOC resources to patients and HCPs, and enhance visibility and access through centralized entry points and online hubs. This is particularly important for provinces without dedicated programs and resources.	Provincial and territorial governments Patient organizations Healthcare providers Hospitals

## Data Availability

Data presented at the symposium are included in this article. No further data sharing is applicable.

## Appendix A. Conference Organization

The 2025 EAOC Symposium was organized by CCRAN, a national patient advocacy group which has broadened its mandate to encompass all tumour types, providing patient and caregiver support, education and advocacy in Canada.

The objectives and agenda of the two-day virtual symposium were overseen by an Expert Steering Committee comprised on the following members:

- Dr. Michael Raphael (Odette Cancer Centre, Sunnybrook Health Sciences Centre, Toronto, ON, Canada)—Chair;
- Dr. Shady Ashamalla (Odette Cancer Centre, Sunnybrook Health Sciences Centre, Toronto, ON, Canada);
- Dr. Tanya Chawla (Joint Department of Medical Imaging, University of Toronto, Mount Sinai Hospital, Toronto, ON, Canada);
- Dr. Mary Jane Esplen (Department of Psychiatry, University of Toronto, Toronto, ON, Canada);
- Dr. Jason Karamchandani (Department of Pathology, McGill University, Montreal, QC, Canada);
- Dr. Stuart Peacock (BC Cancer Research Institute, Vancouver, BC, Canada);
- Mr. Steve Slack (EAOC Patient Expert; Colorectal Cancer Survivor);
- Ms. Robby Spring (EAOC Patient Expert; Breast Cancer Survivor)

## Appendix B. Collaborating Patient Advocacy Groups

**Table A1:** Collaborating Patient Advocacy Groups.

AYA Canada	Immunocompromised People Are Not Expendable
Brain Tumour Foundation of Canada	Inspire Health
Canadian Breast Cancer Network	Leukemia & Lymphoma Society of Canada
Canadian Cancer Survivor Network	Lung Cancer Canada
Canadian Immunocompromised Advocacy Network	My Gut Feeling Stomach Cancer Foundation of Canada
Canadian Organization for Rare Disorders	Myeloma Canada
Childhood Cancer Canada	Pancreatic Cancer Canada
Cholangio-Hepatocellular Carcinoma Canada	Prostate Cancer Foundation Canada
Craig’s Cause Pancreatic Cancer Society	Quebec Cancer Coalition (Coalition priorité cancer au Québec)
GIST Sarcoma Life Raft Group Canada	Young Adult Cancer Canada
HPV Global Action	

## Appendix C. Conference Registrants

The 2025 EAOC Symposium included 501 registrants, representing various stakeholder groups, specifically healthcare professionals, patients, caregivers, industry partners, researchers, and policy-makers. Registrants were from Canada, United States, United Kingdom, Denmark, Jamaica, Pakistan, Romania, China, Finland, Trinidad and Tobago.

## Appendix D. Conference Agenda

The meeting agenda is presented in Table A2. All sessions were held virtually.

**Table A2:** Conference Agenda.

Session	Speakers
**Day 1:** From Gaps To Action: Transforming Early Age Onset Cancer Detection, Diagnosis & Care Through Policy & Innovation ***Moderator:* Cassandra Macaulay,** Chief Research Officer, CCRAN	
Symposium Opening	**Cassandra Macaulay,** Chief Research Officer, CCRAN
Welcome from CCRAN’s President & CEO	**Filomena Servidio-Italiano,** President & CEO, CCRAN **Jessica Dasler,** Stage 4 Colorectal Cancer Survivor; Patient Advocate; CCRAN’s My Lung Mets and My Advocacy Coach
Addressing the Detrimental Impacts of Early Age Onset Cancer: Key Learnings from CCRAN’s 2024 Early Age Onset Cancer Symposium	**Dr. Michael Raphael,** Medical Oncologist, Early Age Onset CRC Cancer Clinic Lead, Odette Cancer Centre, Sunnybrook Health Sciences Centre; Co-Chair, Medical & Scientific Advisory Board, CCRAN
A National Priority: Lowering the Screening Age for Colorectal Cancer	***Moderator:*** **Dr. Michael Raphael,** Medical Oncologist, Early Age Onset CRC Cancer Clinic Lead, Odette Cancer Centre, Sunnybrook Health Sciences Centre; Co-Chair, Medical & Scientific Advisory Board, CCRAN ***Caregiver:*** **Amanda Conlon,** Co-Founder & Executive Director, Circle Back Foundation; Cousin succumbed to Stage IV Cancer ***Panelists:*** **Dr. Aparna Parikh,** Director of CRC Medical Oncology Research & Young Adult Colorectal Cancer Center, Mass General Brigham Cancer Institute **Dr. Darren Brenner,** Armstrong Investigator in Molecular Epidemiology; Associate Professor, Depts. of Oncology and Community Health Sciences; Division Head—Preventive Oncology; Director of Research—Forzani and MacPhail Colon Cancer Screening Centre; Director—Cancer Screening, Detection and Risk Reduction Program, University of Calgary **Dr. Gary Wild,** Clinical Gastroenterologist & Professor of Medicine, McGill University Health Centre **Dr. Usmaan Hameed,** Colorectal Surgical Oncologist; Clinical Lead, GI Cancer Program & Division Head, General Surgery, North York General Hospital
Considering Cancer in Young Adults: Elevating Awareness & Detection Readiness in Primary Care	***Moderator*:** **Dr. Aisha Lofters,** Family Physician, Women’s College Hospital; CIHR-PHAC Applied Public Health Chair; Associate Professor, Department of Family & Community Medicine, University of Toronto ***Patient:*** **Michelle Burleigh,** Acute Promyelocytic Leukemia Survivor; Patient Advocate; Founder & Patient Consultant, The Clarity Lab; Co-Chair, Canadian Immunocompromised Advocacy Network; Co-Chair, Cancer Action Now Alliance ***Panelists:*** **Dr. Paul Dhillon,** Rural Family Physician, Vancouver Coastal Health, BC; 39 Brigade Surgeon, Canadian Armed Forces; Clinical Associate Professor, UBC **Trudy Matwiy,** Master of Nursing/Nurse Practitioner, Family/All Ages; Nurse Practitioner Primary Care Program (NPPCP); Director of Primary Care, Nurse Practitioner Association of Alberta **Joan Heatherington,** Acute Care Gastroenterology Nurse Practitioner, Red Deer Regional Hospital
Catching Cancer Early: Reframing the System Value of Early Detection in Young Adults	***Moderator:*** **Dr. Tanya Chawla,** Associate Professor & Staff Radiologist, Joint Department of Medical Imaging, University of Toronto ***Patient:*** **Laura Floyd,** Stage III NSCLC Lung Cancer Patient; Patient Advocate ***Panelists:*** **Eva Villalba,** Executive Director, Quebec Cancer Coalition, VBHC Expert **Dr. Craig Earle,** Chief Executive Officer, Canadian Partnership Against Cancer **Jennifer Carey,** Manager of National Advocacy, Canadian Association of Medical Radiation Technologists **Samar Saeed,** Clinical Services Manager, Outpatient Oncology & Systemic Therapy, William Osler Health System
Improving Access to Advanced Diagnostics: Comprehensive Genomic Profiling as a Gateway to Personalized Treatment of Metastatic Cancer	***Moderator:*** **Dr. Jason Karamchandani,** Associate Professor, Departments of Pathology, Neurology and Neurosurgery, McGill University; President, Canadian Association of Pathologists ***Patient:*** **Cynthia Mitchell,** Cholangiocarcinoma Patient; Patient Partner and Advocate ***Panelists:*** **Eddy Nason,** Director, Health, Conference Board of Canada **Dr. Robert Grant,** Medical Oncologist, Princess Margaret Cancer Centre, University Health Network **Dr. Megan Mahoney,** Director, Scientific Affairs and Training, BioCanRx **Dr. Alan Spatz,** Professor, Departments of Pathology & Oncology, McGill University; Chief, Department of Clinical Laboratory Medicine, MUHC; Medical Director, Optilab Montreal-MUHC network
Advancements in the Management of Lung Cancer	***Moderator:*** **Dr. Kevin Jao,** Adjunct Professor, Université de Montréal; Hemato-oncologist, Hôpital du Sacré Coeur de Montréal; Co-Chair, Lung Cancer Canada Medical Advisory Committee ***Patient:*** **Yuan Lew,** Stage IVB Lung Cancer Survivor, EGFR Mutation; Patient Advocate ***Clinician Roundtable:*** **Dr. Shaqil Kassam,** Medical Oncologist, Stronach Regional Cancer Centre **Dr. Elizabeth David,** Vascular Interventional Radiologist, Sunnybrook Health Sciences Centre **Dr. Stephanie Snow,** Medical Oncologist, QEII Health Sciences Centre; Professor, Dalhousie University **Dr. Marcelo Cypel,** Surgical Director, Ajmera Transplant Centre, UHN; Surgical Director, UHN ECLS Program; Canada Research Chair, Lung Transplantation; Professor of Surgery, Division of Thoracic Surgery, University of Toronto, University Health Network; Senior-Scientist, Toronto General Research Institute **Dr. Srinivas Raman,** Radiation Oncologist, BC Cancer Vancouver; Clinical Assistant Professor, Department of Radiation Oncology, UBC
Harnessing the Immune System: Breakthroughs in Immunotherapy	***Moderator:*** **Cassandra Macaulay,** Chief Research Officer, CCRAN ***Patient:*** **Eric Hamilton,** Stage IV Colorectal Cancer Patient; Patient Advocate ***Panelists:*** **Dr. Mairi Lucas,** Medical Oncologist, BC Cancer, Surrey; Assistant Clinical Professor, University of British Columbia (UBC) **Dr. Anuradha Krishnamurthy,** Assistant Professor of Oncology, Roswell Park Comprehensive Cancer Center
**Day 2: Beyond The Diagnosis: Elevating Patient & Family Voices To Improve EAOC Outcomes** ***Moderator:* Cassandra Macaulay,** Chief Research Officer, CCRAN	
**Symposium Day 2 Opening**	**Cassandra Macaulay,** Chief Research Officer, CCRAN
Welcome from CCRAN’s President	**Filomena Servidio-Italiano,** President & CEO, CCRAN **Katie Hulan,** Early Age Onset Stage IV ALK Positive Lung Cancer Patient; Lung Health Advocate
Reviewing CCRAN’s Pan-Tumour Patient Survey Findings: Are Early Age Onset Cancer Patient Needs Being Addressed?	**Filomena Servidio-Italiano,** President & CEO, CCRAN **Cassandra Macaulay,** Chief Research Officer, CCRAN **Shalini Gambhir,** Research Officer, CCRAN
Establishing Young Adult Cancer Clinics: A Patient Group Roundtable	***Moderator:*** **Dr. Perri Tutelman,** Assistant Professor, Chair in Transdisciplinary Mental Health, Department of Psychology, University of Calgary; Co-led Canada’s AYA Cancer Priorities in partnership with AYA CAN ***Patient Group Roundtable:*** **Filomena Servidio-Italiano,** President & CEO, CCRAN **Austin Zimmer,** Support Services Manager & Research Coordinator, Prostate Cancer Foundation Canada **Brenda Clayton,** President & Founder, Cholangio-Hepatocellular Carcinoma Canada; Caregiver of Daughter who succumbed to Early Age Onset Cholangiocarcinoma **Dani Taylor,** Manager of Programs, Young Adult Cancer Canada; Stage III Colorectal Cancer (Lynch Positive) Survivor; Patient Advocate **Maureen Elliott,** Senior Manager, Programs and Support, Pancreatic Cancer Canada **Teresa Tiano,** Chair and Co-Founder, My Gut Feeling, Stomach Cancer Foundation of Canada; Stomach Cancer Survivor and a Nine-Time Cancer Survivor **Bukun Adegbembo,** Director of Operations, Canadian Breast Cancer Network **Michele Wright,** Manager, Patient Support Programs, Lung Cancer Canada
Exploring Intimacy, Reproduction, & Fertility in Early Age Onset Cancer Patients	***Moderator:*** **Christopher Mammoliti,** National Patient Programs Manager & Young Adult Cancer Coach, CCRAN; EAOC Patient Expert; Thyroid Cancer Survivor & Late-Stage Colon Cancer Survivor ***Patient:*** **Julia Girmenia,** Stage IV Inflammatory Breast Cancer Patient; Patient Advocate ***Panelists:*** **Dr. Lauren Walker,** Director, Walker Psychological; Adjunct Associate Professor, University of Calgary **Dr. Caitlin Dunne,** Reproductive Endocrinologist, Fertility Specialist & Co-Director, Pacific Centre for Reproductive Medicine (PCRM) **Dr. Trevor Cohen,** Gynecologic Oncologist, Victoria Centre, BC Cancer Agency **Liz Ellwood,** Founder, Fertile Future; Founder, Le Strategies; Co-Founder, Fertility Match Canada; Stage IB2 Cervical Cancer Survivor; Patient Advocate
Managing the Cost of Cancer: Financial Navigation for Early Age Onset Cancer Patients	***Moderator:*** **Stephen Piazza,** Director of Advocacy, Canadian Cancer Society ***Patient:*** **Chantale Thurston,** Board Chair, AYA Can—Canadian Cancer Advocacy; Stage IV Appendix Cancer ***Panelists:*** **Meg Pfeifer,** Psychosocial Oncology Clinician, CancerCare Manitoba **Shannon Lee Simmons,** Certified Financial Planner; Chartered Investment Manager; Founder, The New School of Finance Inc. **Mary Stuart,** Family Nurse Practitioner, AYA Pediatric Cancer Survivorship Program, IWK Health Centre, Halifax, Nova Scotia
Let’s Talk: Exploring the Cancer Experience Through the Lens of Individuals from Marginalized Populations	***Moderator:*** **Dr. Naheed Dosani,** Palliative Care Physician, St. Michael’s Hospital; Founder & Lead, Palliative Education And Care for the Homeless (PEACH), Inner City Health Associates; Medical Director/Health Equity Lead, Kensington Health; Health Equity Advisor, CPAC; Assistant Professor, University of Toronto ***Patient Panelists:*** **Harjeet Kaur,** Stage IV Rare Blood Cancer Survivor; Patient Advocate; Speaker; Co-Founder, Chai and Hope **J. Nadia Headley,** Stage III Breast Cancer Survivor; Patient Advocate; Strategic Director, Twenty One Fourteen Consultancy Services; Executive Director, The Women’s Centre of Halton **Kaylee Engle,** Stage IV Melanoma Patient **Peter Laneas,** Testicular Cancer Survivor (Stage IIIA & IA); Advocate; Advocacy & Engagement Lead, Cancer Fatigue Services
Life Beyond Treatment: Improving the Survivorship Experience	***Moderator:*** **Dr. Mary Jane Esplen,** Psycho-Oncologist; Professor, Department of Psychiatry, Temerty Faculty of Medicine, University of Toronto ***Patient:*** **Robby Spring,** Stage I Breast Cancer, Luminal B, Survivor; Patient Advocate ***Panelists:*** **Dr. Margaret Fitch,** Professor (Adjunct), Bloomberg Faculty of Nursing, University of Toronto **Dr. Stuart Peacock,** Professor and Leslie Diamond Chair in Cancer Survivorship, Simon Fraser University; Distinguished Scientist, BC Cancer **Dr. Lianne Trachtenberg,** Clinical and Health Psychologist, Lianne Trachtenberg Psychology
Closing remarks	**Filomena Servidio-Italiano,** President & CEO, CCRAN
